# Emergency hospital admissions among older adults living alone in the community

**DOI:** 10.1186/s12913-021-07216-3

**Published:** 2021-11-03

**Authors:** Jon Barrenetxea, Kelvin Bryan Tan, Rachel Tong, Kevin Chua, Qiushi Feng, Woon-Puay Koh, Cynthia Chen

**Affiliations:** 1 Health Services and Systems Research, Duke-NUS Medical School Singapore, Singapore, Singapore; 2 Ministry of Health, Singapore, Singapore; 3 Saw Swee Hock School of Public Health, National University of Singapore, 12 Science Drive 2, 117549 Singapore, Singapore; 4 Integrative Sciences and Engineering Programme, NUS Graduate School, National University of Singapore, SG Singapore, Singapore; 5 Department of Sociology & Centre for Family and Population Research, National University of Singapore, Singapore, Singapore; 6 Healthy Longevity Translational Research Programme, Yong Loo Lin School of Medicine, National University of Singapore, 5 Science Drive 2, 117545 Singapore, Singapore; 7 Singapore Institute for Clinical Sciences, Agency for Science Technology and Research, Singapore, Singapore

**Keywords:** Independent living, Hospitalization, Patient admission, Length of stay, Healthcare costs, Chronic disease, Ageing in place

## Abstract

**Background:**

Among older adults, living alone is often associated with higher risk of Emergency Department (ED) admissions. However, older adults living alone are very heterogeneous in terms of health. As more older adults choose to live independently, it remains unclear if the association between living alone and ED admissions is moderated by health status. We studied the association between living alone and ED admission outcomes (number of admissions, inpatient days and inpatient costs) among older adults with and without multimorbidity.

**Methods:**

We used data from 16,785 individuals of the third follow-up of the Singapore Chinese Health Study, a population-based cohort of older Singapore Chinese (mean age: 73(61-96) years). Participants were interviewed face-to-face from 2014 to 2016 for sociodemographic/health factors and followed-up for one year on ED admission outcomes using Singapore Ministry of Health’s Mediclaim Database. We first applied multivariable logistic regression and two-part models to test if living alone is a risk factor for ED admission outcomes. We then ran stratified and joint effect analysis to examine if the associations between living alone and ED admission outcomes were moderated by multimorbidity.

**Results:**

Compared to living with others, living alone was associated with higher odds of ED admission [Odds Ratio (OR) 1.28, 95 % Confidence Interval(CI) 1.08-1.51)], longer inpatient days (+0.61, 95 %CI 0.25-0.97) and higher inpatient costs (+322 USD, 95 %CI 54-591). The interaction effects of living arrangement and multimorbidity on ED admissions and inpatient costs were not statistically different, whereas the interaction between living arrangements and multimorbidity on inpatient days was borderline significant (p-value for interaction=0.050). Compared to those living with others and without multimorbidity, the relative mean increase was 1.13 inpatient days (95 %CI 0.39-1.86) for those living alone without multimorbidity, and 0.73 inpatient days ( 95 %CI 0.29-1.17) for those living alone with multimorbidity.

**Conclusions:**

Older adults living alone were at higher risk of ED admission and higher inpatient costs regardless of multimorbidity, while those living alone without multimorbidity had the longest average inpatient days. To enable aging in place while avoiding ED admissions, interventions could provide instrumental support and regular health monitoring to older adults living alone, regardless of their health status.

## Introduction

Emergency Department (ED) admissions account for 82 % of unplanned hospitalizations and pose a considerable financial and resource burden to healthcare systems [1, 2]. Older adults are more likely to incur ED admissions due to chronic diseases, physical limitations, cognitive impairment and falls [3–7]. As the proportions of older adults increase in the population, hospital resources could be strained due to ED admissions linked to age-related complications [8, 9]. Yet, studies have shown that 46 % of such ED admissions are potentially preventable [10]. A plausible solution to ensure the sustainability of health services is to identify groups of older adults at higher risk of ED admissions. Doing so could help design interventions that fill the gaps in medical care at the community level and prevent older adults from resorting to costly ED admissions [11].

Older adults are increasingly living alone [12], which is associated with a higher risk of ED admissions [13–16], longer inpatient days [17–22] and higher inpatient costs [19]. Since older adults living alone are not able to draw support as immediately as those living with others, they could rely more on acute tertiary services when emergencies arise [23–25]. At the same time, extensions in life expectancy will increase the pool of older adults who live alone with multimorbidity [26]. While multimorbidity is an established risk factor for ED admission outcomes [5, 6, 9, 27–32], it remains unclear if the association between living alone and ED admissions is modified by multimorbidity. This is because older adults living alone are widely heterogeneous in their physical, social and psychological capabilities [33–36]. While some are functionally independent and have no chronic diseases, others may deal with multimorbidity without support, which could lead to further complications and emergency hospitalizations [37–42]. Hence, studying if the association of living alone on ED admissions is modified by multimorbidity could help assess individual risks with more precision.

This is particularly relevant in Asia, where trends towards living alone are gaining momentum in recent years [43]. While living alone in old age is common in Western societies, in Asia, cultural norms of filial piety have traditionally encouraged multigenerational co-residence [44]. As a result, most older adults belong to social networks that are restricted to their adult children, who act as the main care providers for older people [45, 46]. However, as family sizes shrink and the number of older adults increases, sustaining cultural norms of co-residence and caregiving will become more challenging. Older adults are adapting their expectations accordingly by choosing to live independently while receiving ad-hoc financial and instrumental support from their adult children [47]. This could be problematic in societies where state sponsored care services for older adults have not been fully developed due to strong reliance on family as the first line of support [48]. As governments step in to fill the gaps in family support, targeting groups at higher risk of ED admissions could be the first step towards containing healthcare costs.

Singapore is a city-state in Southeast Asia with a rapidly ageing population and shrinking family sizes [49]. In 2019, the proportion of older adults living alone was 22.9 % and the number of older adults living alone is expected to increase fourfold in the next 40 years [50, 51]. The government policies towards the care of older adults follow the so called “many helping hands” framework [46]. In this framework, family is established as the first line of support for older adults, followed by support from the wider community, such as friends, voluntary welfare organizations and other social groups [52]. Government assistance is thus focused on older adults who cannot draw enough support from family [46]. This assistance could include day care services at senior activity centres, befriending activities, subsidies for assistive devices and instrumental help to remain ambulant in the community [53].

We used data from community-dwelling Singaporean older adults to study the association of living alone and multimorbidity on ED admissions. We first examined if living alone is a risk factor for ED admissions, longer inpatient days and higher inpatient costs after adjusting for demographic, health and social factors. We then ran stratified analysis by multimorbidity status to test if the effect of living alone on ED admissions was different between older adults with and without multimorbidity.

## Methods

### Study participants

The Singapore Chinese Health Study (SCHS) is a population-based cohort of 63,275 Singaporean Chinese aged 45–74 years at the time of recruitment (1993-1998). Recruitment for the baseline study included residents from government-built housing estates, where 86 % of Singaporeans resided at that time. The cohort was established initially to study diet and nutrition in cancer aetiology [54]. After the baseline interview, consenting participants were re-contacted for the first follow-up (1999–2004, *N*=52,322), second follow-up (2006–2010, *N*=39,528) and third follow-up (2014–16, *N*=17,107) interviews for updates on factors such as diet, lifestyle and medical history. In addition, the third follow-up included data on ageing outcomes such as cognitive function, instrumental limitations, depression and social support. All participants gave written informed consent. This study was approved by the Institutional Review Board of the National University of Singapore.

In this study, we used data from the third follow-up interviews, which took place from 2014 to 2016 and were conducted by trained interviewers in-person. The initial sample included 17,107 surviving participants with a mean age of 73 years (range: 61-96 years). After excluding participants with missing values on depression tests (*n*=157), cognitive tests (*n* = 55), physical functioning (*n*=2), self-rated health (*n*=2), and participants who were mute (*n* = 1), blind (*n* = 55), deaf (*n* = 48) and living in nursing homes (*n*=3), the final sample included in this analysis was comprised of 16,785 participants (6,854 men and 9,931 women).

### Inpatient hospitalization outcomes

The SCHS cohort was linked with the Mediclaim Database, which is a nationwide database hosted by the Ministry of Health, Singapore, for the purpose of capturing all patient discharge information submitted by accredited institutions, both public and private. It contains records of individual-level inpatient hospitalizations (number of admissions, inpatient days and cost), regardless of whether there are financial claims or otherwise [55]. For our purpose, inpatient hospitalizations were categorized as planned (if the admission was scheduled beforehand) or unplanned (if the participant was admitted through the ED). For this analysis, we only considered unplanned (ED) inpatient hospitalizations as they comprised 70 % of all inpatient admission costs in our cohort. In addition, around 46 % of unplanned inpatient admissions could be potentially prevented through preventive primary care services and timely outpatient care prior to the admission [10]. Hence, understanding the factors associated with higher risk of unplanned admission outcomes could help reduce healthcare costs by targeting groups at risk. Inpatient hospitalization cost was based on total expenditure before government subsidy and insurance. Therefore, the individual out-of-pocket expenditure would be lower after accounting for subsidies and insurance pay-outs. Costs were converted to United States Dollars (USD) based on the average exchange for the year 2016 (1 USD to 1.38 Singaporean Dollars).

### Assessment of living arrangements and multimorbidity

Based on living arrangements, we categorized participants as living alone or living with others. Among those living with others, participants lived either with family (spouse, children or spouse and children) or with other relatives/friends. Multimorbidity was defined as having two or more of the following 12 self-reported chronic diseases: high blood pressure, hypercholesterolaemia/hyperlipidaemia, diabetes, cancer, stroke, heart disease (acute myocardial infarction/angina/heart failure/coronary artery bypass graft or angioplasty), gout, arthritis, bone fracture, chronic lung disease, kidney failure and Parkinson’s disease.

### Other health factors

Due to their association with higher ED admission risk, we included the following health factors as potential confounders in our analysis: physical function, instrumental limitations, mild cognitive impairment and depression. Physical function was defined in our study using items 1 to 3 of EQ-5D-3L questionnaire, which assessed mobility, self-care and usual-activity problems in three levels (no problems, some problems or unable to perform the activity) [56]. Those with no problems across all three items were considered as having ‘good physical function’, whereas those with some problems or unable to perform any of the three tasks were considered as having ‘poor physical function’. We assessed instrumental limitations using the Lawton Instrumental Activities of Daily Living Scale (IADL) [57], which has been validated in Asian populations [58]. We rated each activity dichotomously and computed summary scores from 0 (low function) to 8 (high function). Respondents were classified as those with no instrumental limitations (score of 8) and those with at least one instrumental limitation (score less than 8). Mild cognitive impairment was assessed using a modified Singapore version of the Mini Mental State Examination (MMSE) [59]. Since our study population had relatively low educational level, we defined cognitive impairment using education-specific cut-off points of less than 18 for those without formal schooling, less than 21 for those with 1-6 years of education, and less than 25 for those with more than 6 years of education [60]. We measured depressive symptoms using the Geriatric Depression Scale (GDS) scores and being depressed was defined as having GDS scores equal or higher than 5. The GDS has been validated among community-dwelling older adults in Singapore [61].

### Social factors

Since social factors are enablers of healthcare access and utilization [62], we considered social participation and perceived social support in our analysis. Social participation was assessed with this question: “How many hours each week do you participate in any groups (≥3 people) such as a social or work group, church-connected group, self-help group, charity, public service or community group?”. Those with “zero hour” of social participation per week were considered as having “no social participation”. Perceived social support was measured with the Duke Social Support Scale (DUSOCS). The DUSOCS scale defined perceived social support as “having someone who is helpful, who will listen to you, or who will back you up when you are in trouble” and measured the amount of support in four different levels: “none”, “some”, “a lot” and “there is no such person”. “None” meant that the participant perceived no support from that person, whereas “there is no such person” indicated that such a person did not exist (e.g. parents were dead, participant was not working and thereby had no co-workers, participant had no siblings etc.). The scale identified six sources of family support (partners, children/grandchildren, parents, siblings, other relatives and relatives by marriage) and four sources of non-family support (neighbours, co-workers, religious group members and friends). The questionnaire also included a question on whether there was a main person the respondent can rely on. The scores were then summated to compute the total social support score, which ranges from 0 (no support) to 100 (most support) [63].

### Statistical analysis

The analysis period comprised the year following the interview date at follow-up 3. To model inpatient costs and inpatient days, we used a two-part model. This has become the standard to model over-dispersed outcomes with a large proportion of zero and a skewed distribution [64]. The first part consisted of a probit model that used the full sample to estimate the probability of a participant having any positive value on inpatient costs or inpatient days. The second part comprised of a Generalized Linear Model (GLM) that focused on those participants with positive values on inpatient costs and inpatient days. This second part followed a gamma distribution with log-link for inpatient costs and a negative binomial distribution for inpatient days.

We adjusted the models for age, gender, education level (no formal education, primary school, secondary school and above), health factors, social factors, primary care utilization and ED inpatient admissions for the year preceding the interview date. Because healthcare expenditure tends to increase sharply as individuals approach death [65, 66], we also adjusted for mortality within the analysis period. All statistical analyses were conducted using Stata Statistical Software, Release 14.0 (StataCorp, College Station, Texas) with 2-sided p-value less than 0.05 as the threshold for statistical significance.

In our analytical strategy, we first examined if living alone was a risk factor for ED admission outcomes after adjusting for the aforementioned demographic, health and social factors. Next, we conducted stratified analysis by dividing the participants into two subgroups of multimorbidity (yes, no) to study if the effect of living alone on ED admission outcomes was different between older adults with and without multimorbidity. We assessed the difference in the risk estimates between these two subgroups by computing the p-value for difference using the method described by Altman and Bland [67]. Finally, we assessed the joint effect of living arrangements and multimorbidity on ED admission outcomes by comparing the four categories, namely: (1) living with others and without multimorbidity (reference group), (2) living with others and with multimorbidity, (3) living alone without multimorbidity and (4) living alone with multimorbidity. We then tested for interaction effects between living arrangement and multimorbidity by adding the cross product of living alone and multimorbidity status to the fully adjusted models and extracting the p-values for interaction.

## Results

In our cohort, only 7.8 % of our participants lived alone, and older adults living alone were more likely to be older, female, less educated, less socially supported and more depressed than those living with others. On the other hand, older adults living alone also had less instrumental limitations and more social activity than those living with others. About 62.3 % in the cohort reported having multimorbidity and 15.3 % had four or more chronic diseases. The number of chronic diseases did not differ significantly by living arrangements. Our participants had on average 0.23 ED admissions per year, which lasted on average 1.51 days and amounted to an average cost of 1,159 USD. Considering only those who had any ED admissions (14.5 % of our cohort), the average number of admissions was 1.56, with an average of 10.78 inpatient days and an average inpatient cost of 8,009 USD (Table 1).

**Table 1:** Characteristics of study participants according to living arrangement status, the Singapore Chinese Health Study [n (%) and mean (standard deviation)]

	Total (*N*=16,785)	Living with others (*N*=15,473)	Living alone (*N*=1,312)	p-value
Number of ED admissions, mean (SD)	0.23 (0.69)	0.22 (0.69)	0.28 (0.74)	<0.01
Excluding those with no ED admissions	1.56 (1.11)	1.56 (1.12)	1.54 (1.07)	0.80
Inpatient days, mean (SD)	1.51 (7.08)	1.47 (7.07)	2.03 (7.17)	<0.01
Excluding those with <1 day	10.78 (16.04)	10.70 (16.30)	11.52 (13.56)	0.46
Inpatient costs (USD), mean (SD)	1,159 (5,480)	1,139 (5,482)	1,390 (5,464)	0.11
Excluding those with no costs	8,009 (12,363)	8,038 (12,518)	7,728 (10,834)	0.71
Age (years), mean (SD)	73.2 (6.40)	73.0 (6.37)	75.0 (6.46)	<0.01
Gender, n (%)				<0.01
Men	6,854 (40.8)	6,548 (42.3)	306 (23.3)	
Women	9,931 (59.2)	8,925 (57.7)	1,006 (76.7)	
Education level, n (%)				<0.01
Secondary education and above	6,114 (36.5)	5,702 (36.8)	412 (31.4)	
Primary education	7,509 (44.7)	6,930 (44.8)	579 (44.1)	
No formal education	3,162 (18.8)	2,841 (18.4)	321 (24.5)	
Number of chronic diseases, n (%)				0.30
None	2,697 (16.1)	2,506 (16.2)	191 (14.6)	
One	3,629 (21.6)	3,348 (21.6)	281 (21.4)	
Two	4,304 (25.6)	3,977 (25.7)	327 (24.9)	
Three	3,587 (21.4)	3,288 (21.2)	299 (22.8)	
Four or more	2,568 (15.3)	2,354 (15.3)	214 (16.3)	
Multimorbidity, n (%)				
Without multimorbidity (None or one chronic disease)	6,326 (37.7)	5,854 (37.8)	472 (36.0)	0.18
With multimorbidity (Two or more chronic diseases)	10,459 (62.3)	9,619 (62.2)	840 (64.0)	
Physical function, n (%)				0.04
Good physical function	14,686 (87.5)	13,561 (87.6)	1125 (85.7)	
Poor physical function	2,099 (12.5)	1912 (12.4)	187 (14.3)	
Instrumental limitations (Lawton), n (%)				<0.01
None	12,318 (73.4)	11,295 (73.0)	1,023 (78.0)	
At least one	4,467 (26.6)	4,178 (27.0)	289 (22.0)	
Depression (GDS score ≥5), n (%)				<0.01
Not depressed	12,343 (73.6)	11,458 (74.1)	885 (67.5)	
Depressed	4,442 (26.4)	4,015 (25.9)	427 (32.5)	
Cognitive impairment (MMSE), n (%)				0.79
Not cognitively impaired	14,363 (85.6)	13,244 (85.6)	1,119 (85.3)	
Cognitively impaired	2,422 (14.4)	2,229 (14.4)	193 (14.7)	
Social support, n (%)				<0.01
Adequate social support	15,029 (89.5)	14,014 (90.6)	1,015 (77.4)	
Poor social support	1,756 (10.5)	1,459 (9.4)	297 (22.6)	
Social activity, n (%)				<0.01
Socially active	8,865 (52.8)	8,046 (52.0)	819 (62.4)	
Not socially active	7,920 (47.2)	7,427 (48.0)	493 (37.6)	
Primary care visits, n (%)				
Not in highest decile	14,990 (89.3)	13,808 (89.2)	1,182 (90.1)	0.34
Highest decile	1,795 (10.7)	1,665 (10.8)	130 (9.9)	
Baseline number of ED admissions, mean (SD)	0.16 (0.53)	0.16 (0.53)	0.17 (0.52)	0.69
Excluding those with no ED admissions	1.38 (0.88)	1.39 (0.89)	1.35 (0.78)	0.58
Baseline inpatient days, mean (SD)	0.89 (4.61)	0.89 (4.63)	0.93 (4.33)	0.76
Excluding those with <1 day	8.03 (11.60)	8.05 (11.72)	7.89 (10.27)	0.87
Baseline inpatient costs, mean (SD)	950 (5,233)	954 (5,320)	903 (4,077)	0.74
Excluding those with no costs	8,238 (13,321)	8,317 (13,620)	7,365 (9,396)	0.39
Died within analysis period				0.51
No	16,431 (97.9)	15,150 (97.9)	1,281 (97.6)	
Yes	354 (2.1)	323 (2.1)	31 (2.4)	

In multivariable analysis, compared to living with others, living alone was associated with a higher odds of ED admission [Odds Ratio (OR) 1.28, 95 % Confidence Interval (CI) 1.08-1.51)], longer inpatient days (+0.61, 95 % CI 0.25-0.97) and higher inpatient costs (+322 USD, 95 % CI 54-591). In addition, higher number of chronic diseases was associated with higher ED admission risk, longer inpatient days and higher inpatient costs in a stepwise manner (p for trend<0.001). Compared to those without multimorbidity, those with multimorbidity had higher odds of ED admission (OR 1.30, 95 % CI 1.17-1.44), longer inpatient days (+0.30, 95 % CI 0.06-0.54) and higher inpatient costs (+360 USD, 95 % CI 199-522) (Table 2).

**Table.2:** The association of living alone and number of chronic diseases on ED admission outcomes

	ED admission risk [OR (95 % CI)]°	Inpatient days [Mean increase (95 % CI)]°	Inpatient costs (USD) [Mean increase (95 % CI)]°
Living arrangements			
Living with others	1.00 (Ref.)	0.00 (Ref.)	0.00 (Ref.)
Living alone	1.28** (1.08 - 1.51)	0.61*** (0.25 - 0.97)	322** (54 - 591)
Number of chronic diseases			
None	1.00	0.00 (Ref.)	0.00 (Ref).
One	1.23* (1.03 - 1.46)	0.27 (-0.08 - 0.62)	25 (-211 - 262)
Two	1.30** (1.10 - 1.53)	0.13 (-0.18 - 0.43)	163 (-80 - 406)
Three	1.44*** (1.22 - 1.71)	0.47** (0.11 - 0.83)	301* (41 - 561)
Four or more	2.21*** (1.86 - 2.63)	0.98*** (0.59 - 1.37)	814*** (494 - 1,134)
p-for trend	<0.001	<0.001	<0.001
Multimorbidity			
Without multimorbidity (Less than two chronic diseases)	1.00	0.00 (Ref.)	0.00 (Ref).
With multimorbidity (Two or more chronic diseases)	1.30*** (1.17-1.44)	0.30* (0.06-0.54)	360*** (199-522)

°Adjusted for age, gender, education, chronic diseases (hypertension, coronary artery disease, stroke, diabetes, hyperlipidaemia/hypercholesterolaemia, gout, cancer, chronic lung disease, hip/bone fractures, arthritis, Parkinson’s disease and kidney failure), physical function, instrumental limitations, depressive symptoms, mild cognitive impairment, social support, social activity, primary care visits, ED admission outcomes for the year preceding interview date and mortality within analysis period. Those living with others (reference group) had on average 1.48 inpatient days and mean inpatient cost of 1,155 USD. Those with no chronic diseases (reference group) had on average 1.19 inpatient days and mean inpatient cost of 928 USD. Those with no multimorbidity (reference group) had on average 1.36 inpatient days and mean inpatient cost of 964 USD

*p-value<0.05; **p-value<0.01 ***p-value<0.001

In stratified analysis by multimorbidity status, we found that among those without multimorbidity, living alone was associated with longer inpatient days (+0.76 95 %CI 0.21-1.31) and higher inpatient costs (+365 USD 95 %CI 18-712) but did not result in higher odds of ED admissions (OR 1.32 95 %CI 0.97-1.79). On the other hand, among those with multimorbidity, living alone was associated with higher odds of ED admission (OR 1.26 95 %CI 1.03-1.54) and longer inpatient days (+0.50 95 %CI 0.03-0.98) but did not result in higher inpatient costs (+249 USD 95 %CI -119-615). Overall, we found that the effect of living alone on the number of ED admissions, inpatient days and inpatient costs was not statistically different between older adults with and without multimorbidity (p-values for difference between the two subgroups for all three estimates ≥ 0.48) **(**Table 3**)**.

**Table 3:** The effect of living alone on ED admission outcomes among older adults, stratified by the presence of multimorbidity

	ED admission risk [OR (95 % CI)]°	Inpatient days [Mean increase (95 % CI)]°	Inpatient costs (USD)° [Mean increase (95 % CI)]°
Without multimorbidity			
Living with others (*n*=5,854)	1.00 (Ref.)	0.00 (Ref.)	0.00 (Ref.)
Living alone (*n*=472)	1.32 (0.97-1.79)	0.76** (0.21-1.31)	365* (18-712)
With multimorbidity			
Living with others (*n*=9,619)	1.00 (Ref.)	0.00 (Ref.)	0.00 (Ref.)
Living alone (*n*=840)	1.26* (1.03 – 1.54)	0.50* (0.03 - 0.98)	249 (-119 – 615)

°Adjusted for age, gender, education, physical function, instrumental limitations, depressive symptoms, mild cognitive impairment, social support, social activity, primary care visits, ED admission outcomes for the year preceding interview date and mortality within analysis period. Among those without multimorbidity, participants living with others (reference group) had on average 0.99 inpatient days and mean inpatient cost of $680 USD. Among those with multimorbidity, participants living with others (reference group) others had on average 1.77 inpatient days and mean inpatient cost of $1,414 USD

*p-value<0.05; **p-value<0.01 ***p-value<0.001

In joint analysis, we used older adults living with others and without multimorbidity as the reference group to estimate the increased risk of ED admissions, inpatient days and inpatient costs for older adults living alone without multimorbidity and older adults living alone with multimorbidity. Compared to those living with others and without multimorbidity, the odds of ED admission increased by 35 % for those living alone without multimorbidity (OR 1.35, 95 % CI 1.00-1.82) and 64 % for those living alone with multimorbidity (OR 1.64, 95 % CI 1.33-2.03). As for inpatient days, compared to older adults living with others and without multimorbidity, those living alone without multimorbidity had the highest mean increase in inpatient days (+1.13 days 95 % CI 0.39-1.86) followed by those living alone with multimorbidity (+0.73 days 95 % CI 0.29-1.17). As a result, the total increase in hospitalization expenditure was similar for older adults living alone with or without multimorbidity: Compared to those living with others and without multimorbidity, those living alone without multimorbidity had a mean increase of 555 (95 % CI 74-1,036) USD whereas those living alone with multimorbidity had a mean increase of 567 (95 % CI 230-906) USD. While there was no interaction effect between living arrangements and multimorbidity on the risk of ED admission and inpatient costs (p-values for interaction ≥ 0.13), the interaction between living arrangement and multimorbidity on inpatient days was borderline significant (p-value for interaction = 0.050) **(**Table 4**).**

**Table 4:** The joint associations between living alone and multimorbidity (two or more chronic diseases) on ED admission outcomes

	ED admission risk [OR (95 % CI)]°	Inpatient days [Mean increase (95 % CI)]°	Inpatient costs (USD) [Mean increase (95 % CI)]°
Living with others and with no multimorbidity (*n*=5,854)	1.00 (Ref.)	0.00 (Ref.)	0.00 (Ref.)
Living with others and with multimorbidity (*n*=9,619)	1.31*** (1.17 - 1.46)	0.31** (0.10 - 0.53)	353*** (205 - 501)
Living alone with no multimorbidity (*n*=472)	1.35* (1.00 - 1.82)	1.13** (0.39 - 1.86)	555* (74 - 1,036)
Living alone with multimorbidity (*n*=840)	1.64*** (1.33 - 2.03)	0.73** (0.29 - 1.17)	567*** (230 - 906)

°Adjusted for age, gender, education, physical function, instrumental limitations, depressive symptoms, mild cognitive impairment, social support, social activity, primary care visits, ED admission outcomes for the year preceding interview date and mortality within analysis period. Those living with others and with no multimorbidity (reference group) had a mean inpatient length of stay of 1.27 days and mean inpatient cost of 920 USD

*p-value<0.05; **p-value<0.01 ***p-value<0.001

## Discussion

In this study among Singaporean older adults, we found that living alone was associated with higher odds of ED admission, longer inpatient days and higher inpatient costs than living with others. Furthermore, while older adults living alone were at higher risk of ED admissions and higher inpatient costs regardless of multimorbidity, those living alone without multimorbidity were at higher risk of longer inpatient days than their counterparts living alone with multimorbidity.

The results are in line with previous studies showing that older people living alone are at higher risk of ED admissions [13–15, 24]. Older adults living alone are a highly heterogeneous group that also includes those who prefer to live without company, are functionally independent and have an extended network of support to tap into. It has been reported that older adults living alone tend to have more diverse networks of support, while those living with others often have less social ties beyond family [45]. In fact, good health is often a precondition for older adults to live independently, making those living alone more likely to be healthier. In line with this, reverse causality and a wide range of social and psychological confounders could affect the associations between living alone and health outcomes [68]. However, the associations between living arrangements and health follow different mechanisms from the associations between living arrangements and emergency admissions. While we could expect older adults in poor health to be more likely to be hospitalized, the hospitalization route could differ by living arrangements: those living with family could be more likely to have planned admissions whereas those living alone may have more frequent emergency admissions. For example, a healthy older adult living alone could be at higher risk of an emergency admission in the event of a fall compared to another adult in poorer health who lives with a ‘gatekeeper family’ for such events. Moreover, for those living alone without the timely consultation and assistance from family members, the reasons for emergency admissions could go beyond health issues, including poor access to preventive care services, poor health behaviors, lack of health monitoring or lack of instrumental support.

Therefore, regardless of health status and social engagement, older adults living alone may not be able to draw support as immediately as those living with others. This immediate availability of support not only may avert costly ED admissions, but also provide regular assistance in managing age related conditions and prevent further complications [69]. Studies have indeed shown that having informal assistance at home can improve the management of chronic diseases and reduce the need for unplanned hospitalizations and ED visits [23, 70–74]. We also found that older adults living alone are more likely to have longer inpatient days after adjusting for demographic, health and social factors. This suggests that having informal support at home could indirectly shorten inpatient days by ensuring post-discharge assistance.

The fact that multimorbidity did not moderate the association between living alone and risk of ED admission in this study highlights the commonalities of older Singaporeans living alone. Regardless of health status, those living alone tend to pride themselves on self-reliance and not being a burden for their families [47]. The remarkable resourcefulness, coping mechanisms and self-determination of older Singaporeans who live alone has been well documented [25, 47, 75]. This resilience and adaptation to old age adversities could buffer the influence of health problems on healthcare use and explain why multimorbidity status is not an aggravating factor in the association of living alone and ED admission risk.

Interestingly, we found that, compared to those living with others and without multimorbidity, the relative mean increase in inpatient days was 1.13 days for those living alone without multimorbidity and 0.73 days for those living alone with multimorbidity. While we would expect older adults living alone with multimorbidity to have the longest inpatient days, they may also be more health aware and fearful of emergency health expenditures and thus avoid longer hospitalizations related to traumatic injury such as falls [75]. Conversely, those without multimorbidity could be less risk averse and engage in more hazardous activities leading to longer hospitalizations [76, 77]. In addition, those living alone with multimorbidity may interact more often with tertiary care providers and thus be better monitored than their counterparts without multimorbidity. In fact, older Singaporeans who live alone with multimorbidity prefer using tertiary care services for continuity of care because of their established relationship with regular doctors at the hospital [25]. This familiarity could result in more frequent ED admissions related to multimorbidity complications but shorter inpatient days because the case is well known to care providers.

The conclusions presented here also call for adapting existing theories of healthcare use to the Asian population, where living arrangements and family support are key elements of the care continuum of older adults. In the Western literature, the individual typically comes into contact with healthcare systems through primary care for screening purposes or after becoming ill or suffering an injury [78]. In this context, the Andersen’s Behavioral Model of Health Services Use posits that social resources such as availability of family or friends are enabling factors that facilitate access to healthcare services [62]. However, in Asian countries where older adults are heavily supported by co-residing family, living arrangements may not just be an enabling factor but the first point of care for older adults. Co-residing family could therefore act as a gatekeeper of costly ED admissions by monitoring health status, ensuring medical adherence and facilitating access to primary care services. In the absence of support from other household members, older adults living alone could be less aware of their health, have worse adherence to medication regimes and have difficulties accessing primary care services on their own [37–40]. Ultimately, this could aggravate age related complications and result in emergency situations requiring immediate medical care [69]. This pattern of resorting to acute tertiary services due to poorly managed chronic diseases could be even more salient in countries with fragmented primary care and support services for older people.

The results presented here hold policy relevant implications in terms of health service planning amid ageing demographics. Although living arrangements may be hard to modify, the delivery of healthcare can certainly be adapted to a rapidly growing number of older adults lacking informal support at home [42]. Previous research has shown that strengthening primary care services at the community level could fill the gaps in informal support and prevent ED admissions [10, 79, 80]. These services could be complemented with more frequent community nurse visits to monitor health status and manage chronic diseases. Our results also suggest that extending informal support after hospitalization discharge could potentially reduce inpatient days and inpatient costs, particularly among those living alone without multimorbidity. Previous research has also shown that programs facilitating the transition between hospital and community are effective in reducing future re-admissions [11, 81]. In addition, interventions should consider the heterogeneity of older adults and discern between social and medical needs. For example, those living with family may have their instrumental support covered but lack social engagement with people beyond their household, while those living alone may be socially active but lack instrumental support to manage their chronic diseases. As a result, community interventions could fill the gaps in support through befriending activities for those living with family, while providing instrumental assistance to those living alone.

The strengths of this study are the use of individual-level inpatient admission records, the wide range of hospitalization outcomes covered, the large sample size and the use of validated instruments to adjust for physical, social, cognitive and depressive factors among older adults. There are, however, some limitations. First, medical history was based on self-reported data, which is not equivalent to a clinical diagnosis. Similarly, the use of screening instruments such as the GDS, Lawton IADL or the MMSE, while useful for population-based studies, is not equivalent to in-depth clinical assessments. Second, our analysis is an under-estimate as it does not include data from specialist outpatient clinics, which comprise a large fraction of tertiary healthcare expenditure. Third, our independent variables were measured at a fixed point of time and therefore our analysis cannot capture changes in health status and healthcare needs over time. Fourth, the individual-level records could not single out preventable unplanned admissions, which are more likely to be affected by living arrangements than non-preventable admissions. Hence, future research should focus on understanding the social factors influencing preventable unplanned admissions to promote preventive care services among groups at risk.

## Conclusions

We found that older adults living alone were at higher risk of ED admission and higher inpatient costs regardless of multimorbidity, while those living alone without multimorbidity had the longest mean inpatient days. To enable aging in place while avoiding ED admissions, interventions should provide instrumental support and regular health monitoring to older adults who live alone, regardless of their health status. This conclusion could be extended to other Asian countries where older adults have traditionally relied on family support but are increasingly living alone. The results presented here could guide government efforts to fill gaps in family support among older adults living alone and enhancing primary care services at the community level.

## Data Availability

The data that support the findings of this study are available from the Singapore Ministry of Health but restrictions apply to the availability of these data, which are confidential and so are not publicly available. Data are however available from the authors upon reasonable request and with permission of the Singapore Ministry of Health.

## Abbreviations

CI: Confidence Interval
DUSOCS: Duke Social Support Scale
ED: Emergency Department
GDS: Geriatric Depression Scale
GLM: Generalized Linear Model
IADL: Instrumental Activities of Daily Living
MMSE: Mini Mental State Examination
OR: Odds Ratio
SCHS: Singapore Chinese Health Study
USD: United States Dollar
